# Representation and the Voluntary System

**Published:** 1913-09-13

**Authors:** 


### REPRESENTATION AND THE VOLUNTARY SYSTEM.

### A Share in the Management as a Bait for Income.

Dr. T. Basil Rhodes is a believer in the voluntary hospitals, and opposed to the State hospitals, and pleads for their preservation. It is necessary, he states,* that those interested in voluntary hospitals (1) must work, (2) that they must see that their hospitals are properly equipped, and (3) that they must make the management of the voluntary hospitals thoroughly representative. To none of these requirements can any objection be taken. Work is undoubtedly necessary for success of any kind, and it is also undoubtedly necessary that to do good work you must have the proper tools. Nor can much be urged against representative management in the abstract, if by representative management Dr. Rhodes means management by men, desirous of helping the hospital and possessing the necessary knowledge and experience for the purpose, selected from all classes of society. A doubt, however, arises when we come to consider his method for obtaining a committee of this character. Briefly put, it is: "If you will subscribe to us we will make you sharers in the management." Does this bribe really mean anything, and, if so, is it likely to produce men of the character desired? If it means no more than the share, which each voter, for example, has in the management of the Navy, then I take it that those appealed to would no more subscribe to a hospital than they would pay taxes unless they were compelled. They pay taxes because it is the law of the land, and they acquire thereby a right to share in the management of their country by means of their vote; but taxes and gifts are two very different things. People give to a hospital not because they desire some share in its management, but because they wish to do something to help their poorer brethren. Management in itself is a necessary evil.

### The Art of Management and Local Interests.

The only object of a hospital is to cure the sick. Every penny spent on any other is matter for regret. Unfortunately, management cannot be done without; but, so long as the funds of the hospital are properly spent and the patients are treated with skill and kindness, the less the cost of management the better. Theoretically, if the right man could be found, management by one man would be the best; but, human nature being what it is, imperfect so far as one man is concerned and distrustful so far as the subscribing public is concerned, a committee of some sort is necessary. Certainly it is better that this committee should be composed of men or women interested in their work; but it does not follow that the larger the committee, even of interested people, the better do they manage the hospital. Committees of management, in short, are not training grounds; they are or ought to be composed of men who have a knowledge of their work. It is, of course, sometimes unfortunately necessary to conciliate some important local interest by offering a seat on the committee to this or that individual. Luckily, too, it also often happens that this individual turns out an excellent committeeman, but, unless the majority of the committee are sound business men, the hospital suffers. A hospital committee of the average kind is composed of twenty or thirty of the local business men of position, strengthened, as some think, by members of the honorary staff. A committee of this sort may become sleepy and old-fashioned unless it has an energetic chairman to keep it alive, but on the whole it makes no gross errors of management; it is not guilty of fussy interference, and it knows the necessity of discipline. It raises its funds in the old-fashioned way by appealing to the charitable public. It has a yearly deficit of so many thousands and it trusts to a large legacy every ten or a dozen years to put its accounts right. A good chairman is able to get all the help he requires from his committee; an indifferent chairman is overwhelmed with too much advice, and the committee drifts helplessly along.

No doubt like every other human institution it has its weaknesses, and the introduction of a little new blood every now and then is all for its good. By all means let it leaven its mass by the introduction of representatives of the working-classes, and so long as the number of these is kept within bounds it will benefit; but it must be prepared for difficulties, and one of the difficulties is that the more money the working-classes raise, the more representation they require on the committee of management.

### Dangers of Co-operative Management.

Now it is here that the danger of using the bait of a share in the management as a means of raising money comes in. It is not difficult to do, and by means of it sums of money sufficient to keep a hospital large enough for the needs of the district can be obtained, provided, that is, that only those are allowed admission as patients who cannot afford the treatment they require. This, however, is not all. You have promised a share in the management, and you will find that the larger the amount raised from the work-people the more representatives on the committee of management they will require. The old-fashioned committee of management may be something of a close corporation and may want wakening up; but there is such a thing as overdoing it. A point is reached when lack of trust becomes ridiculous. If the public cannot trust a committee of thirty, do not let them imagine that they are going to improve matters by doubling it. In all probability all they are going to do is to double the distrust. Nor does it stop here. The governors themselves begin to make their voices heard. They are possessed with this idea of management, and at their quarterly courts they endeavour, by bringing forward motions

* *The North Staffordshire Infirmary: Recent History and Present Condition, with Additional Chapters on Voluntary Hospitals in General.* By T. Basil Rhodes, M.B., B.S. (Secretary and House Governor).

on this and that subject, to take out of the hands of their executive work which only their executive is properly qualified to deal with, and before long the hospital is converted into something that resembles a co-operative store. Let those who advocate co-operative management, for that is what it comes to, try the experiment of dealing at co-operative stores and find out for themselves whether they get goods of better quality at lower prices, or are served with more attention and more quickly than at the old-fashioned shops.

A further danger which seems inseparable from this form of government is that the representatives of the governors are not really trusted by the men who appoint them. The representatives themselves are taught to believe that they are the delegates, that is, they lack independence. They are apt to vote on matters not so much from the point of view of the good of the institution as from the way in which their vote will be regarded by those who elected them. They are anxious to be re-elected, and they do not care to take any step they think might render them unpopular. Very often they are men who know nothing whatever of hospital management, who have to be educated in their job, and once they have become really useful members of the committee they unfortunately sometimes fail to gain re-election, and the process of education has to be gone over afresh, to the detriment of the hospital. With a strong chairman difficulties can be tactfully overcome, but it makes the work of managing a hospital more difficult rather than easier; to put it quite bluntly, the real management of a hospital has to be done " in spite of " not " by means of " their assistance. Where the body of governors is very large and the number of their representatives on the committee is proportionate to their numbers, there is often fussy interference with subordinate officials, nurses, etc., and discipline is undermined. This is the darker side of the picture. On the other hand, the representatives of the working-people of the district are very often men who are keenly interested in their work and really anxious to help. They are able to bring the needs of the hospital home to the people in their district; they are often able to prevent unnecessary complaints ever being brought forward; they are able to keep an eye that cases of gross abuse of charity do not occur; they are able to bring before the committee in an unanswerable manner the patient's point of view, and this is a most extremely important thing; they are often men of capacity and character and make most excellent members of committee, and if a good case for any reform in the hospital can be made out to them, they are among the first to back it up.

The quarrel is not with the introduction of working-men on to the committee of management. They make just as good members of committee as men from any other class. The quarrel is with this bribe of management, which is addressed to men who, naturally enough perhaps, misunderstand it and who take it too seriously. Interference is not management, however well meant that interference may be. Nor is it management to elect men on to your committee who are delegates without minds of their own. Nor is it management to form your committee with any men except those whose one object is to cure the sick.

You will not get men of proved ability to give their time to the work if they are to be harassed with petty interference, or if their time is to be wasted by colleagues who have no knowledge of the work, and who sit on the board for every other object under the sun than the one and only object of helping to cure those who are sick. You cannot expect men to sit and listen to the arguments of Socialists, or of the co-operator who wishes all goods to be bought from co-operative societies, or of the trade-union man who conceives it his duty to force all men who wish to conclude contracts with the hospital to pay trade-union rate of wages, and so on. Very estimable objects all of them, possibly, in their proper place; the mistake is that a hospital committee of management is not the proper battlefield on which to fight for them. By all means spread interest in the hospital as widely as possible, but do not let it be at the expense of its management. Let your committee be composed of men from different classes—business men, professional men, and workmen—but let it be of moderate size, and, when you have chosen it, let it have a free hand to act according to its lights, and let the public judge by results.

### The Motive of Charity.

Lastly, there is a danger connected with contributions made from any other motive than that of charity pure and simple. The working-men are so accustomed to deductions for this or that society, which give them the right to this or that benefit, that they cannot help regarding their contribution to a hospital as carrying with it the right to treatment irrespective of their ability to pay for it themselves. They have a difficulty in understanding that their contribution is a charitable one to help those who are less fortunate than themselves; and though, of course, the majority of working-men are unable to pay for most operations, yet there are operations which they could, and which they formerly did, pay for. In this matter there is, however, opportunity for education, and there would soon be no reason why it should not be possible to organise committees in each district to safeguard the hospital from abuse, to guarantee, in short, that the patients coming from those districts are really suitable for charitable relief. So far as the out-patients are concerned, the abuse of this department is usually by the workmen who live in the immediate neighbourhood of the hospital. Workmen who live at a distance naturally cannot make use of it to the same extent, and as these latter contribute the same amount as the town dwellers, they may very naturally argue that as they cannot use the hospital out-patient department for their minor ailments, neither is it fair that the town dwellers should. The committee of management is thereby supplied with a lever to prevent all workmen coming to the hospital from getting treatment which they can perfectly well afford to get outside.

